# Applications of Molecular Markers for Developing Abiotic-Stress-Resilient Oilseed Crops

**DOI:** 10.3390/life13010088

**Published:** 2022-12-28

**Authors:** Vishal Chugh, Dasmeet Kaur, Shalini Purwar, Prashant Kaushik, Vijay Sharma, Hitesh Kumar, Ashutosh Rai, Chandra Mohan Singh, Kamaluddin, R. B. Dubey

**Affiliations:** 1 Department of Basic and Social Sciences, College of Horticulture, Banda University of Agriculture and Technology, Banda 210001, India; 2 Department of Environmental and Plant Biology, Ohio University, Athens, OH 45701, USA; 3 Department of Basic Sciences, College of Forestry, Banda University of Agriculture and Technology, Banda 210001, India; 4 Instituto de Conservación y Mejora de la Agrodiversidad Valenciana, Universitat Politècnica de València, 46022 Valencia, Spain; 5 Department of Genetics and Plant Breeding, College of Agriculture, Banda University of Agriculture and Technology, Banda 210001, India; 6 Department of Genetics and Plant Breeding, Maharana Pratap University of Agriculture and Technology, Udaipur 313001, India

**Keywords:** molecular markers, MAS, oilseeds, abiotic stress, SSRs, molecular breeding, climate change

## Abstract

Globally, abiotic stresses, such as temperature (heat or cold), water (drought and flooding), and salinity, cause significant losses in crop production and have adverse effects on plant growth and development. A variety of DNA-based molecular markers, such as SSRs, RFLPs, AFLPs, SNPs, etc., have been used to screen germplasms for stress tolerance and the QTL mapping of stress-related genes. Such molecular-marker-assisted selection strategies can quicken the development of tolerant/resistant cultivars to withstand abiotic stresses. Oilseeds such as rapeseed, mustard, peanuts, soybeans, sunflower, safflower, sesame, flaxseed, and castor are the most important source of edible oil worldwide. Although oilseed crops are known for their capacity to withstand abiotic challenges, there is a significant difference between actual and potential yields due to the adaptation and tolerance to severe abiotic pressures. This review summarizes the applications of molecular markers to date to achieve abiotic stress tolerance in major oilseed crops. The molecular markers that have been reported for genetic diversity studies and the mapping and tagging of genes/QTLs for drought, heavy metal stress, salinity, flooding, cold and heat stress, and their application in the MAS are presented.

## 1. Introduction

All annual oilseed crops have experienced poor growth rates over the previous ten years (negative for area and production), particularly safflower, sunflower, linseed, and niger crops and especially peanut, which has also experienced negative growth for the area [1]. India is the world’s largest importer of vegetable oils (15 percent market share), followed by China and the USA, and it relies heavily on imports to meet its edible oil needs [2]. Palm oil accounts for roughly 60% of all imported edible oils, followed by soybean oil (~25%) and sunflower oil (~12%). It is also projected that edible oil demand will be 40.9 Mt by 2026 and that, by 2050, India will need to generate 17.84 Mt of vegetable oils to satisfy the country’s estimated 1685 million population [2].

The necessity to scale up oilseed production demands immediate attention, given the rising domestic need for edible oils, alarming shortage, and the expense on the exchequer resulting from imports. Strategies to increase the productivity (and profitability) of oilseed-based production systems include the development of abiotic-stress-tolerant varieties in the context of changing climatic conditions. Increased frequency of extreme events (floods, cold, droughts, heat, etc.), altered precipitation patterns, and an increase in average temperature (average high night temperature) are all indicators of climate change. As Earth transitioned between ice ages over the last 800,000 years, atmospheric concentration of CO_2_ fluctuated between 180 ppm (glacial times) and 280 ppm (interglacial eras). The CO_2_ concentration has steadily increased from pre-industrial levels of 280 ppm to 384 ppm in 2009, while the mean temperature has risen by 0.76 °C over that time. According to projections, atmospheric [CO_2_] will reach 700 ppm or more by the end of this century, while the global temperature will rise by 1.8–4.0 °C depending on the greenhouse emission scenario [3]. With regard to global climate models, the mean ambient temperature is predicted to further increase by 1.5 °C within the next two decades [4]. The abiotic stresses have been reported to cause moderate to severe yield loss in various oilseeds (Table 1). To develop abiotic stress resistance and thus increase oil yield per unit area, traditional breeding efforts must be amalgamated with biotechnology methods.

**Figure 1:**
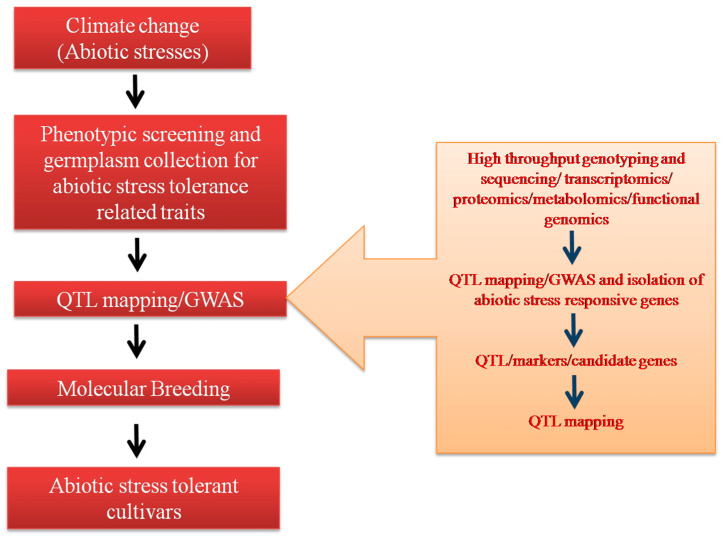
A stepwise presentation of molecular breeding and genomics approaches for the development of abiotic-stress-tolerant cultivars.

Molecular markers such as RFLP (Random Amplified Polymorphic DNA), AFLP (Amplified fragment length polymorphism), SSR (Simple Sequence Repeat), RAPD (Random Amplified Polymorphic DNA), SNP (single nucleotide polymorphisms). etc., are DNA-based oligonucleotide sequences facilitating the detection of variations or polymorphisms in the population for specific regions of DNA. Among the various biotechnological interventions, molecular markers have played a pivotal role in accelerating the crop breeding programs employing marker-assisted selection (MAS). The facts that they are abundant, technically easy to use, and detectable at any stage of plant development have given them an added advantage of not being affected by environmental factors. Rapid advancements have been made in the development of a variety of molecular markers over the past 20 years with refinements on a regular basis depending on the available infrastructure, technical skills required, the importance of the crop, and the trait in question. Figure 1 illustrates how molecular and integrated plant breeding is helpful in developing varieties with abiotic stress tolerance using genomic approaches such as MAS [23]. Success has been achieved in breeding oilseeds, such as canola, mustard, sunflower, soybean, and peanut, through the utilization of molecular marker techniques, mapping traits that control seed quality, and biotic and abiotic stress resistance [24,25,26,27,28,29,30]. However, though the techniques and available tools for MAS are well established, there is still a dearth of studies conducted using MAS to achieve abiotic stress tolerance for edible oilseed crops, such as sesame, niger, safflower, and the non-edible oil crops castor and linseed. This article discusses case studies involving the use of molecular markers for developing abiotic-stress-tolerant cultivars/genotypes of various oilseed crops.

## 2. Applications of Molecular Markers in Development of Abiotic-Stress-Tolerant Oilseed Crops

### 2.1. Drought

**Figure 2:**
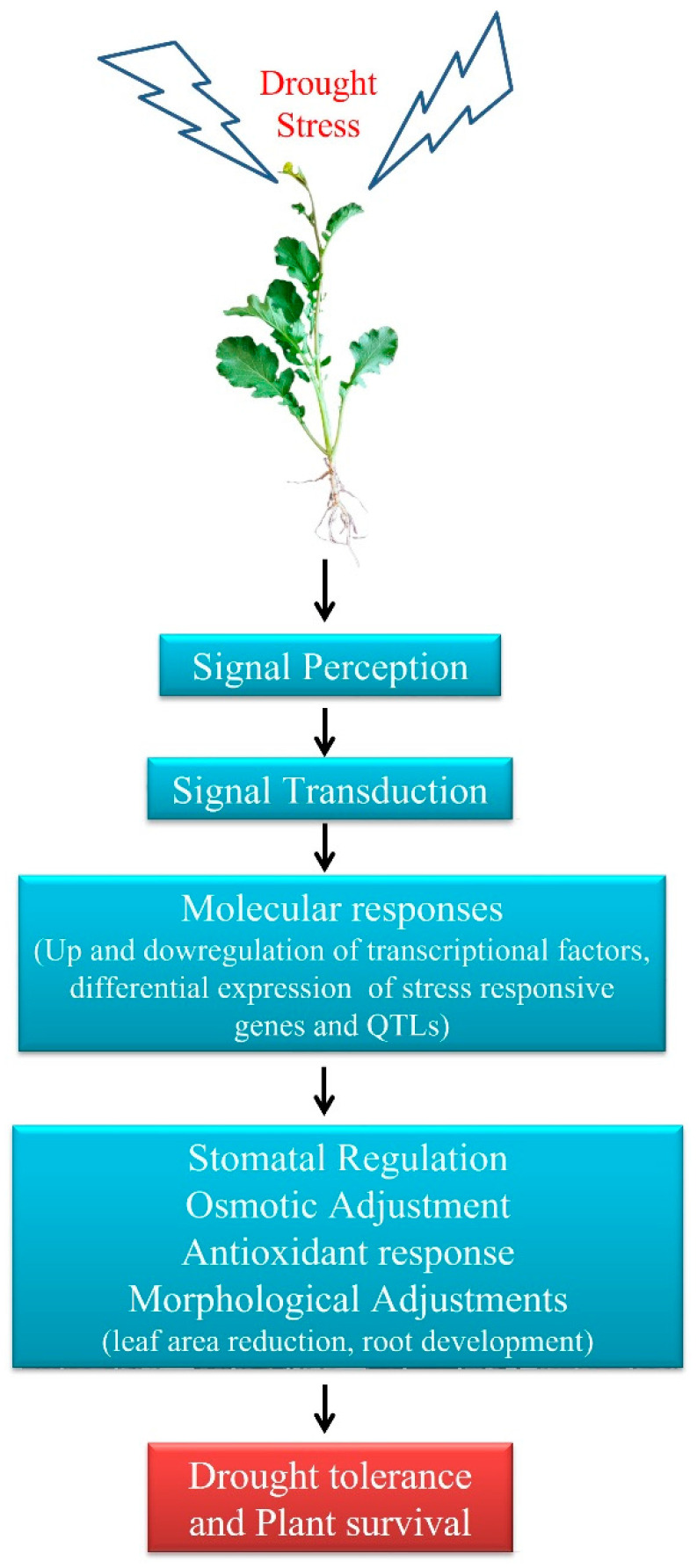
Cellular morphological and molecular responses in plants help to combat drought stress.

Fresh water scarcity is an emerging global problem. Since agriculture primarily harnesses freshwater, enhancing agricultural output amid restricted water availability is a major challenge [31]. Although improvements in irrigation and tillage methods can be used to conserve water and increase crop yield, supplementary strategies like genetic modification of crops are required for increasing productivity under moisture deficiency conditions [32,33]. Estimates indicate that adverse environmental factors affect about half of the possible crop production, with water shortage being the most severe stress [34,35,36]. Tolerance to drought is a quantitative attribute that is influenced by numerous genes via a variety of mechanisms in a plant. Under drought stress, expression patterns in genes that are involved in water transport; osmotic balance; oxidative stress; morphological modifications, including root development and reduced leaf area; and damage repair are altered (Figure 2).

A number of studies have provided deeper insights into understanding the molecular basis of drought tolerance in plants [37,38,39,40]. Drought alters the growth, physiology, and metabolic activities of plants, which in turn have an adverse impact on the nutritional quality and yield of important oilseed crops around the world [41,42]. In drought stress, it has been observed that plants’ enzymatic activity is reduced, which eventually penalizes yield and quality of oilseeds [43]. Under conditions of water deficiency, a decrease in the oil content of soybean seeds has been reported [44]. Genomic resources created using various methods, such as genotyping-by-sequencing (GBS), genome sequencing, genome-wide association studies (GWAS), etc., have given researchers strong tools for characterizing the genetic diversity of oilseed crops, a solid framework for finding new traits, and next-generation breeding tools to speed up the development of elite cultivars. Comparative genome analysis is one of the significant advantages of the current growth of genomic data.

Numerous quantitative trait loci (QTLs) for traits associated with physiological, agronomic, seed composition, and abiotic and biotic stress parameters have been reported in soybean *(Glycine max*) [24,45]. Only a small number of QTLs have, however, so far been linked to characteristics related to drought resistance. Additionally, reported QTLs account for 10% or less of the phenotypic variance for those traits. To date, the majority of research focusing on the identification of QTLs has used small, single populations. Chen et al. [46] discovered QTLs associated with primary root length on chromosome 16 of soybean, which accounts for 30.25% of the variation in phenotype and will help in the development of markers for root-length selection, which is a crucial trait for drought tolerance. In 1996, a research team led by Mian developed an RFLP map in soybean from a population of 120 F_4_-derived lines of a cross ‘Young × PI416937’ that identified the multiple QTLs that are associated with leaf ash and water use efficiency (WUE) [47]. For both attributes, authors reported significant (*p* < 0.01) differences at the phenotypic level among the lines. In total, four and six independent RFLP markers were reported to be linked with the said two traits, respectively, and when added together, each set of markers would be responsible for 38 and 53% of the variance in the corresponding traits. A significant QTL was found at marker position cr497-1 on USDA Linkage Group (LG) J, which accounted for 13.2% of the variability in WUE. The scientists also noted that two QTLs were linked to both WUE and leaf ash and that leaf ash and WUE had a negative correlation (r = −0.40). One QTL associated with RFLP marker A063E for WUE was also detected in the ‘Young × P1416937’ population; however, the phenotypic effect was merely <10%, according to authors who tested another soybean population derived from F_2_ progenies developed from the cross of ‘S100 × PI41693 [25]. To date, only WUE and leaf ash QTLs have been documented in soybean under water deficit conditions. More extensive research is required in order to find QTLs that affect shoot turgor maintenance and root architecture. Finding novel QTLs and genes, as well as deciphering the mechanism governing how genes behave during drought, could prove to be hugely instrumental in enhancing soybeans’ ability to withstand drought stress.

Numerous genes likely associated with drought tolerance have been identified in sunflower (*Helianthus annuus* L.), including *HaDhn1* (sunflower dehydrin gene)*, SunTIP* (sunflower tonoplast intrinsic protein), *HaDhn2*, *Sdi* (sunflower drought induced), *Hahb-4* (sunflower homeobox-leucine zipper gene)*,* and *HAS1* (sunflower, asparagine synthetase) or *HAS1.1*. These genes have been reported to exhibit high levels of expression under drought stress, and it has been speculated that they contribute to the tolerance of sunflower to drought stress [26,48,49,50]. However, only a handful of studies on sunflower have been conducted to ascertain the development of molecular markers for QTLs linked to drought tolerance [27]. Hervé et al. [28] employed the AFLP linkage map to recognize QTLs for water status (transpiration and leaf water potential), stomatal movements, and net photosynthesis. Using the AFLP linkage map, 19 QTLs were identified, which accounted for 8.8–62.9% of the phenotypic variance for each characteristic. Out of these, two significant QTLs for net photosynthesis were found on linkage group IX [28]. Similar to this, 24 QTLs were discovered in sunflower in well-watered conditions, of which 5 (or around 21%) were also discovered following drought condition. A range of 6% to 29% of phenotypic variance was explained by the QTLs [51].

Safflower (*Carthamus tinctorius* L.) mapping, molecular breeding, and QTL discovery pronouncedly lag behind other oilseed crops due to a lack of genetic data [52]. As a result, there has been very little genetic enhancement of safflower through marker-assisted breeding and linkage of characteristics. In 2010, Tang et al. mapped heat shock protein (HSP) genes by utilizing a cDNA–AFLP linkage study with 192 randomly segregating F_2_ populations [53]. Genomic and EST-SSR markers, which can be useful for mapping, molecular breeding, and the linkage of desirable QTL traits like drought tolerance, have been developed in safflower by a number of research groups [52,54]. In this direction, an intra-specific F_2_ population of *Carthamus tinctorius* and an inter-specific BC_1_ population of *Carthamus tinctorius* × *Carthamus oxyacanthus* were mapped by generating 1142 PCR based markers and 75 RFLP markers to undertake the first major linkage study of the *Carthamus* species. Both of these mapping populations’ utilized these EST-SSR markers [55]. Another researcher noted the feasibility of transferring non-genic microsatellite (SSR) markers and gene-based markers from sunflower (*Helianthus annuus* L.) to safflower. These markers comprised resistance gene candidates (RGC)-based markers and intron fragment length polymorphism (IFLP) [56]. In F_3_ families produced from the hybrid of the tolerant Mex.22-191 (tolerant) and sensitive IL.111 (sensitive) safflower genotypes under drought stress, QTLs linked to seed yield and its attributes were mapped using SSR and ISSR markers [57]. This study discovered 18 QTLs linked to seed yield and its attributes, including four major QTLs and three linkage groups (2, 4, and 6), which were found to be crucial for safflower’s ability to withstand drought.

In spite of large morphological variation observed between germplasm accessions, peanut (*Arachis hypogaea* L.) shows very little genetic variation at the molecular level, as detected by markers like isozymes, RFLPs, and RAPDs [58]. Three independent research groups around the world have invested in the development of microsatellite markers for peanut and have reported up to 200 simple sequence repeats (SSRs) [29,59,60]. About 20% of them can detect peanut polymorphism. Moreover, a genetic map of 191 SSR loci was constructed based on a single mapping population (TAG 24 × ICGV 86031) segregating for drought and surrogate traits [61]. The QTL Cartographer identified 105 significant impact QTLs (M-QTLs) explaining 3.48 to 33.36 percent of the phenotypic variance (PVE), but the QTL Network only identified 65 M-QTLs that explained 1.3 to 15.0 percent of the PVE. Comparing the two programmes together allowed the identification of 53 common M-QTLs. Additionally, genotype matrix mapping (GMM) identified 186 (8.54–44.72% PVE) and 63 (7.11–21.13% PVE) three and two loci interactions, respectively, while only 8 epistatic QTLs (E-QTL) interactions with 1.7–8.34% PVE were identified by the QTL network. This study led the authors to conclude that the discovery of some major and many minor M-QTLs and QTL × QTL interactions underpinned the complex and quantitative nature of drought tolerance in peanut. It was recommended that genomic selection or marker-assisted recurrent selection be used as a breeding strategy for drought tolerance instead of marker-assisted backcrossing [61]. In another related study, a screening of two RIL (recombinant inbred lines) mapping populations, viz., ICGS76 × CSMG84-1 (RIL-2) and ICGS44 × ICGS76 (RIL-3) with 3215 SSR markers, two genetic maps with 119 (RIL-2) and 82 (RIL-3) SSR loci were constructed. Using these aforementioned maps based on two RIL populations and a reference map of 191 SSR loci based on the TAG 24 × ICGV 86,031 RIL population, Gautami et al. [62] constructed a dense consensus map of 293 SSR loci distributed across 20 linkage groups, spanning 2840.8 cm. In addition to a total of 153 M-QTL and 25 E-QTL for drought tolerance, the authors reported the discovery of 16 prospective genomic regions carrying 125 QTL related to biomass, yield, and drought component traits. In summary, this study identified many QTLs with low to moderate phenotypic variance for the complex traits such as biomass, yield, and drought tolerance. These studies potentially provided a direction for additional investigation and exploitation for QTL pyramiding and cloning in the future, though the discovery of major QTL/s for drought tolerance is still awaited.

Sesame is a hardy crop that is well-adapted to drought prone areas. Sesame typically endures drought better than other important food crops [63]. The production of this oil-rich crop is, however, still quite sensitive to droughts that occur during the germination and flowering stages [64,65]. Unfortunately, there are only a few molecular-marker-based studies conducted so far deciphering the genomic regions associated with sesame’s tolerance under drought conditions. Dossa et al. [66] conducted a GWAS employing SNP markers for variables interrelated to drought tolerance in 400 different sesame accessions, including landraces and potential modern varieties. This study reported 10 stable QTLs associated with drought-tolerance-linked characteristics located in four linkage groups. Additionally, this study reported two significant pleiotropic QTLs harboring both known as well as unknown genes for drought tolerance, such as *SiTTM3* (*Sesamum indicum* Triphosphate tunnel metalloenzyme 3), *SiABI4* (*Sesamum indicum* ABA insensitive 4)*, SiGOLS1* (*Sesamum indicum* Galactinol synthase 1), *SiNIMIN1* (*Sesamum indicum* NIM1-Interacting 1), and *SiSAM* (*Sesamum indicum S*-adenosylmethionine synthetase). In order to identify candidate genes associated with drought tolerance in the whole genome of sesame, researchers conducted a comparative homology search with three relative species, viz., potato, tomato, and *Arabidopsis* [67]. The authors successfully identified 75 candidate genes (42, 22, and 11 from *Arabidopsis*, potato, and tomato, respectively), which were found to be distributed on the 16 sesame linkage groups. Based on their functional classification, authors divided the genes in two groups. One group consisted of genes that protect the plant against drought effects, while the other included signal transduction genes and transcription factors. Several other studies have also employed molecular markers for QTL mapping and GWAS to unravel the genetic basis of drought tolerance in sesame [68,69,70,71,72].

Although we have witnessed remarkable progress in the field of genomics over the last ten years, the availability of precise and high-throughput phenotyping for drought tolerance traits is still a major challenge for QTL mapping studies. Targeting root architecture, photosynthetic efficiency, osmotic adjustment, relocation of stem reserves, and leaf senescence under drought stress are among the phenotypic features that could benefit the most from the application of MAS. Further, the construction of consensus maps integrating the QTL information provided by different populations needs more attention. It is certain that molecular-assisted breeding has the potential to more effectively address the problems caused by the diminishing availability and rising cost of irrigation water, as well as the escalating demand for food, fiber, and biomass.

### 2.2. Salinity

**Figure 3:**
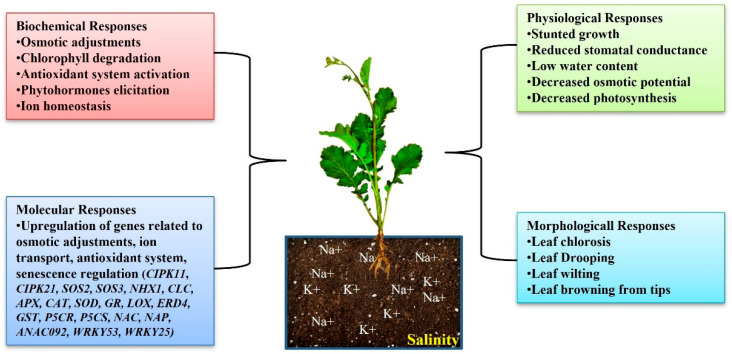
Plant responses to salinity stress. [Abbreviations: *CIPK*: CBL-interacting protein kinases; *SOS*: salt overly sensitive; *NHX*: sodium/hydrogen antiporter; *CLC*: chloride channel; *APX*: ascorbate peroxidase; *CAT*: catalase; *SOD*: superoxide dismutase; *GR*: glutathione reductase; *LOX*: lipoxygenase; *ERD*: early responsive to dehydration; *GST*: glutathione S-transferase; *P5CR*: pyrroline-5-carboxylate reductase; *P5CS*: pyrroline-5-carboxylate synthetase; *NAC*: NAC transcription factor; *NAP*: nucleosome assembly protein; *ANAC*: *Arabidopsis* NAC transcription factor; *WRKY*: WRKY transcription factors].

One of the key abiotic stress challenges influencing the quality and production of food crops globally is soil salinity, which restricts crop plants’ growth and development [73,74]. Furthermore, salinity can pose risks to the production of oilseeds by lowering both the yield and quality of the produce. Globally, salt affects >833 million hectares of land [75], and it is believed that 20% of cultivated and 33% of irrigated land are affected [76]. By preventing cell division, enzyme activity, nucleic acid and protein synthesis, and salinity stress negatively impacts seed germination and seedling growth, height, leaf size, leaf number, reproductive structures, seed quantity, seed content, seed weight, and the quality of seed oil [30,31,32,33,34,35,36,37,38,39,40,41,42,43,44,77,78,79,80,81,82]. Figure 3 illustrates how a plant also responds at biochemical, molecular, physiological, and morphological levels to salinity stress in order to sustain its growth and production [83]. However, decades of intensive research have led to the improved comprehension of the mechanisms by which salt stress affects crop development and productivity. Indeed, this information may be used to develop genotypes that are salt-tolerant.

Numerous factors, including soil properties, genotypes, and developmental phases, influence how oilseed crops react to salt stress. Although the majority of oilseed species are prone to damage under salt stress, nevertheless a wide range of diversity in terms of salt sensitivity exists among them. While canola, soybean, sunflower, and safflower exhibit moderate to strong tolerance, peanut and linseed are examples of sensitive species [84]. Likewise, it has been observed that amphitetraploid *Brassica* species, such as *B. juncea, B. carinata,* and *B. napus,* are relatively more tolerant against salt stress compared to their progenitors, such as *B. nigra*, *B. rapa*, and *B. oleracea*. Among all the *Brassica* species, *B. napus* is extremely tolerant to salt stress, whereas *B. rapa* and *B. nigra* are extremely sensitive [85]. Since tolerance to salt stress is a physiologically intricate trait, the development of salt-tolerant genotypes necessitates a comprehensive approach that involves modifying existing cultivars genetically and biotechnologically.

An essential method for localizing the genomic areas that regulate characters related to salt stress tolerance is QTL mapping. QTLs are identified using powerful DNA marker approaches, such as AFLP, RFLP, RAPD, SSR, and SNPs. Successful breeding for salt stress in oilseeds, notably in *Brassica* species, requires the identification of QTL. It is challenging to detect a genetic basis for salinity tolerance in *Brassica* species, since no significant QTL with relation to salinity tolerance in those species has yet been discovered due to the physiological complexity of the salinity response. However, a limited number of studies have demonstrated the utilization of molecular markers in this field. RFLP markers that were used to characterize each line to find salt-tolerance-related QTL in soybean RILs produced S-100 (tolerant cultivar to salinity) and a Tokyo variation (susceptible cultivar to salinity). After that, a single-factor QTL analysis was performed to discover trait-related genomic areas. To improve mapping accuracy, specific genomic areas were flooded with SSR markers. The study found a QTL related to salt tolerance at SSR marker Sat 091 at LG N. In the field, greenhouse, and mixed environments, this QTL was found to be responsible for 41%, 60%, and 79% of salt tolerance, respectively. In fact, the tolerance-related QTL alleles were found to be derived from S-100 through pedigree tracking [86]. Using two RIL populations resulting from the cross between FT-Abyara C01 and Jindou No. 690197, a similar study discovered a substantial salt-tolerance QTL in soybean’s molecular linkage group N. This study employed FT-Abyara C01 and Jindou No. 690,197 RIL populations. This QTL accounted for 44.0% to 47.1% of salt tolerance across the two groups [87]. Using a separate linkage group, Chen et al. [88] found a second significant QTL (qppsN.1) between markers Sat 164 and Sat 358 on linkage group G in a cross of Kefeng No.1 (salt-resistant) and Nannong 1138-2 (salt-sensitive) soybeans.

In similar research, Hamwieh and Xu [89] discovered a QTL related to salt-tolerance in soybean on linkage group N with a substantial dominant impact from 225 lines of F_2_ population produced from a cross of Jackson (PI548657) (salt-resistant cultivar) × JWS156-1 (salt-sensitive wild soybean). This major QTL explained 68.7% of the variance in the salt tolerance rating scale. The authors concluded that both wild and cultivated soybeans carry the conserved QTL related to salt tolerance, which has a significant dominating effect over salt sensitivity.

Most widely used markers in safflower are ISSRs, AFLPs, and RAPDs because they are ideal for crops with little genetic resources, require no prior knowledge, and perform genome scanning with repetitive sequences [90]. Safflower genetic diversity has been documented in numerous investigations employing a combination of phenotypic variation and molecular polymorphism [91,92,93].

In 2018, Li and his co-researchers used a diversity panel of 490 accessions of sesame (*Sesamum indicum*) to conduct a genome-wide analysis of stress tolerance indices related to sodium-chloride-induced salt stress and PEG-induced drought stress to understand the resulting genetic variants with respect to drought and salinity tolerance at the germination stage [68]. According to this study, under the stresses of drought and salt, respectively, there were 132 and 120 significant SNPs, which further resolved to be associated with 9 and 15 QTLs. There were just two shared QTLs for the response to salt and drought, which were situated in the linkage groups (LGs) 5 and 7, respectively. Authors also reported a total of 13 and 27 potential candidate genes for drought and salt tolerance indices, respectively, which encode transcription factors, osmoprotectants, and antioxidant enzymes and are associated with signal transduction, hormone biosynthesis, or ion sequestration, which were also reported for the drought and salt tolerance indices, respectively.

In an attempt to elucidate the genesis of wild sunflower hybrid’s (*H. annuus × H. petiolaris*) adaptation to salt stress, Lexer et al. [94] employed EST markers on 11 genes. One EST was mapped to QTL responsible for salt tolerance, which encodes a Ca-dependent protein kinase (*CDPK*) that originated in stress-induced root tissue of *H. annuus;* hence, a plausible adaptive role for Ca-dependent salt tolerance genes in wild sunflower hybrids was suggested. Another study by the same author on 172 BC_2_ hybrids between *Helianthus annuus* and *Helianthus petiolaris* planted in the salt marsh habitat of *Helianthus paradoxus* in New Mexico identified 14 QTLs for mineral ion absorption attributes and three for survivability [95]. The previous results that suggested that salt tolerance in Helianthus is achieved through higher Ca^+^ absorption, along with stronger exclusion of Na^+^ and similar mineral ions, were confirmed by mineral ion QTLs mapping to the same place as the survival QTLs (on LG 1, 4, and 17b). In a separate study, researchers evaluated the variability of microsatellites from genomic areas that were neutral in the experimental hybrids with that of microsatellites associated with the three survival QTLs listed above. It was established that populations of the natural hybrid species had significantly less variability according to microsatellites relating to the survival QTLs. However, in parental populations, there was no discernible difference in the levels of diversity between the two microsatellite classes [96].

With the above information in mind, it is evident that considerable effort has been put into identifying the genes or QTLs that contribute to salinity tolerance in oilseed crops; nevertheless, there are presently few reports of cultivars or breeding lines with better salt tolerance that have been successfully developed using molecular markers and MAS technology. The limited use of markers for improving complex traits like salinity tolerance has been attributable to various reasons; however, it is possible to use markers for such complex traits by the identification of reliable QTLs and linked genetic markers. This can be achieved by putting additional efforts, such as conducting mapping experiments in field conditions instead of a greenhouse, so that plants experience actual salt stress in association with other stresses and environmental factors as well, studying the cross-tolerance mechanism that exist in plants against various stresses; the identification of QTLs in multiple environments; splitting complex traits, such as salt tolerance, into individual components; and identifying QTLs and markers for such individual components instead of studying salt tolerance as a whole (such as finding QTLs for salinity tolerance at different developmental stages), and finally the pyramiding of such QTLs may pave the way to develop salt-tolerant oilseed crops. This is a challengeable but achievable strategy to follow in order to develop salt tolerance in plants. Table 2 summarizes some of studies that have used molecular markers in the development of resistance to abiotic stresses in oilseed crops.

### 2.3. Heavy Metal Stress

Heavy metal poses a global concern because of its significant technological implications in several industrial processes and applications. Different heavy metals from these sectors that severely contaminate wastewater have numerous long-term ecological and biological harmful impacts [116]. The heavy metals in this wastewater are hazardous, and if this discharged water is used for irrigation purposes, it disturbs the biological balance of the soil and the plants that are grown there. Since heavy metals are naturally prevalent in the earth’s crust, they can be found in both polluted and unpolluted soils. Synthetic fertilizers, contaminated sewage/sludge, manure, and mining and industrial operations can all cause heavy metals to emerge in agricultural soil [117]. When sewage water is added to the soil, plant growth may increase, but it may also include toxic substances that may threaten crops and the food chain. Many heavy metals, including Mo, Fe, Ni, Cu, Mn, and Zn, are advantageous or necessary for plant growth in low concentrations, but in high concentrations, they are all toxic [117]. In particular, a heavy dosage of these metals may cause oxidative stress, inhibit root elongation, displace other essential metals in a plant’s enzymes, introduce pigments that cause the function of many metabolic processes to be disrupted, and ultimately compromise the yield and growth [118,119,120]. Heavy metals are proven to be toxic for oilseed crops in a variety of ways, and the symptoms vary greatly depending on the plant, metal, and its dose [121]. Oilseeds have the ability to reduce toxicity caused by metals optimizing their hemostasis. In oilseeds, heavy metals promote free radicals’ generation, compete with metal cofactors of plant enzymes, alter enzyme action through binding sulfhydryl and N-containing groups, and cause the leakage of cellular contents through interactions with phospholipid-containing groups. *Brassica* cultivars have demonstrated decreased plant growth caused by Pb toxicity through altered cellular metabolism and nutrient uptake [122]. In fact, under Cr stress, such negative effects were also seen in shoot growth, leaf area, and length of leaf [123].

In order to facilitate MAS in RIL population (F_6:8_) obtained from a cross of AC Hime (high-Cd accumulation) × Westag-97 (low-Cd accumulation)’ soybean, Jegadeesan et al. [100] conducted a study to develop markers for low-Cd accumulation. It was demonstrated by the use of 171 SSR markers that low-Cd accumulation in soybean seeds is regulated by a key gene (*Cda1*), with the low-accumulation allele being dominant. In soybean seeds, *Cda1* was found to be associated with 7 SSR markers, viz. SatK138, SatK139, SatK140, SatK147, SacK149, SaatK150, and SattK152. Each linked marker was assigned the same linkage group K. Markers SatK147, SacK149, and SattK152 distinguished studied genotypes with low and high Cd accumulation. Additionally, a significant QTL linked to a low Cd level in seeds was mapped to the same region at linkage group K as *Cda1*. This QTL was identified as the source of 57.3% of the phenotypic variation [100]. It has been experimentally proven that molecular markers can be used to locate particular loci regulating soybean resistance to Mn toxicity [101]. In a previous study, researchers demonstrated that RAPD markers could identify four QTLs, or hotspots, in an RIL population descended from the “Essex × Forrest” cross that may be responsible for resistance to manganese toxicity [124]. However, a study was conducted by using only high-quality scores generated by 240 microsatellite markers to detect the QTL that underlie tolerance to Mn toxicity in the F_5_-derived RIL population from “Essex × Forrest” (E × F, *n* = 100). The study was performed in order to rule out the errors occurring in RAPD maps and consequent errors in assigning QTL [101]. The necrosis of the leaves and roots served as markers. The findings showed that root necrosis at 7 days after treatment was strongly linked (*p* ˂ 0.005, R2 = 20%) with the regions on linkage groups I (BARC Satt239), C2 (BARC Satt291), and G (OP OEO2); these three QTLs could explain about 58% of the total variation in root resistance to Mn toxicity. They also affirmed one of the previously identified RAPD-associated root necrosis QTLs, namely, sudden death syndrome QTL on LG (G). However, no QTL for leaf chlorosis were identified (*p* ˂ 0.005), and none of the RAPD-associated with leaf chlorosis QTL could be confirmed [101].

### 2.4. Flooding

**Figure 4:**
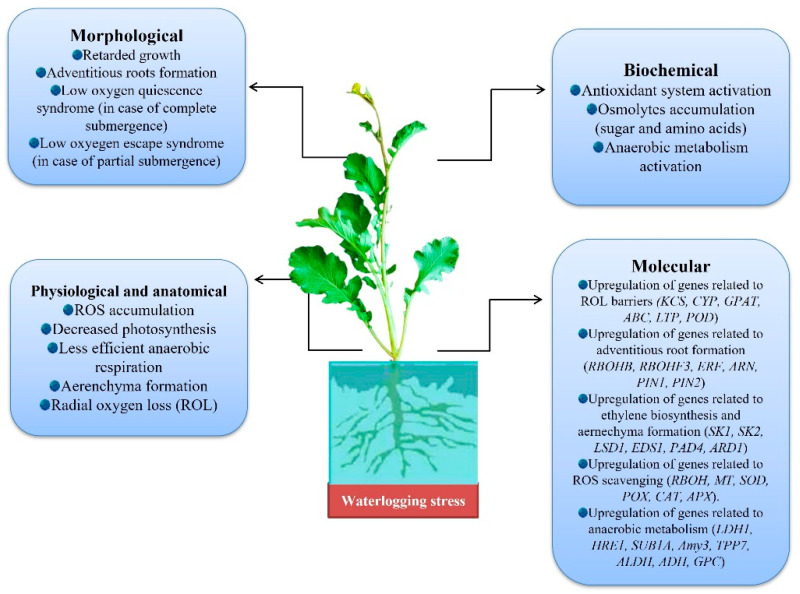
Response and the adaptive mechanisms of plants under flooding stress. [Abbreviations: *KCS*: 3-ketoacyl CoA synthase; *CYP*: cytochrome P450; *GPAT*: glycerol-3-phosphate acyltransferase; *ABC*: ATP-binding cassette; *LTP*: lipid transfer protein; *POD*: peroxidase; *RBOHB*: respiratory burst oxidase homolog B; *ERF*: ethylene response factor; *PIN*: PIN formed proteins; *SK1*: SNORKEL1; *SK2*: SNORKEL1; *LSD:* lesion simulating disease; *EDS*: enhanced disease susceptibility; *PAD*: phytoalexin deficient; *ARD:* acireductone dioxygenase; *MT*: metallothionein; *SOD:* superoxide dismutase; *POX*: peroxidise; *CAT*: catalase; *APX*: ascorbate peroxidise; *LDH*: lactate dehydrogenase; *HRE*: hypoxia response element; *SUB1A:* submergence tolerance regulator; *Amy3:* α-amylases; *ADH*: alcohol dehydrogenase.

The main barrier to sustainable agriculture is flooding, and the plants exposed to flooding experience significant yield losses. Plants frequently encounter intermittent or persistent floods in their natural habitat. Physio-chemical soil characteristics that are crucial include redox potential, soil pH, and oxygen content, which are altered by flooding in a variety of ways. As a result, plants growing in wet soil endure stressful conditions, such as hypoxia (a lack of oxygen) or anoxia (the absence of O_2_). These low oxygen environments have a significant negative impact on plant growth, development, and survival. Metabolic changes under oxygen deprivation, including switching to anaerobic respiration and oxidative damage caused by reactive oxygen species (ROS), compromise membrane integrity, as well as damage photosystem II’s efficiency, leading to a significant decline in net photosynthetic rates. To combat flooding-induced hypoxia/anoxia and oxidative stress, plants that endure waterlogging stress have mechanisms, such as the better availability of soluble sugars, formation of aerenchyma, enhanced glycolysis and fermentation activity, and the contribution of antioxidant defense mechanisms [125,126]. Many flooded plant species, including soybean, have shown evidence of developing adventitious roots [127,128,129]. Figure 4 illustrates a variety of responses and coping mechanisms used by plants to cope with flooding stress.

It has now become simple for scientists to focus on altering or using the key genes that have been linked to flooding tolerance, to eventually develop new flood-tolerant plant varieties. Genomic areas linked to flooding tolerance can be detected using map-based gene cloning and QTL mapping. In the case of rice, it was able to introduce the *Sub1* gene to particular varieties by molecular-assisted backcrossing (MAB) to accommodate a different soil type and farmer preferences, as well as to add new variations through genetic engineering [126]. In an effort to map QTLs conferring flooding tolerance in soybean, two hundred and eight lines of two RIL populations descending from the ‘Archer × Minsoy and Archer × Noir I’ were placed in two different experimental setups: one under controlled condition (no flooding) and the other under flooding condition (waterlogging). Plants were subjected to 2 weeks of flooding at the early flowering stage in a water-logged setup, in order to identify the QTL linked to soybean flooding tolerance. Authors discovered a single QTL from the Archer parent, associated with marker Sat 064, that was responsible for the increased growth of plants (11 to 18%), as well as seed yield (47 to 180%), in a flood environment. Both RI populations included this highly significant QTL (*p* = 0.02–0.000001). Authors also reported that Sat_064 QTL on Chromosome 18 was distinctively linked with flooding tolerance and was not linked with normal plant length, maturity, or seed yield. Although the Rps4 gene and Sat 064-QTL are co-localized for resistance to *Phytophthora sojae* resistance, the donor parent Archer lacks the Rps4 resistance allele, proving that Sat 064-QTL is exclusive for flooding-stress tolerance [102]. Further, this QTL was verified in NILs at the F_6_ generation descended from heterogeneous inbred families [105]. Tolerant NILs produced around 60.9% greater yields under stress-free conditions compared to the yield of sensitive NILs (32.6%) under the same environment. Using bulked segregant analysis (BSA), as well as partial linkage mapping, two more QTLs concerning flooding-tolerance traits were also identified and were found to be linked to markers Satt385 on Chromosome 5 and Satt 269 on Chromosome 13 [106]. The advantageous alleles of these two QTLs came from Archer.

In another investigation, 60 RILs of soybean were derived from cross ‘Misuzudaizu (flooding tolerant cultivar) × Moshidou Gong-503 (flooding sensitive cultivar)’ in order to study the genetics of tolerance to flooding stress at early vegetative stage. The plants were grown in pots and were subjected to flooding treatment at the two-leaf stage for 3 weeks. Pots were then put back in the greenhouse to mature there. The experiment was conducted for two consecutive years. In 2002, three QTLs for flooding tolerance, *ft1* to *ft3*, were identified, employing 360 genetic markers. Four other QTLs, numbered *ft4* to *ft7*, were discovered in 2003 in addition to the *ft1* (linkage group C2), which was reproducible. In both years, *ft1* possessed a high LOD (logarithm of the odds; relative probability that two loci are linked) score (15.41 and 7.57) and contributed 49.2% and 30.5%, respectively, of the overall variance. At a location identical to *ft1*, a major QTL for days to blooming was seen across all treatments and years [103]. It was further observed that the main QTL caused a prolonged recovery period prior to the reproductive stage by delayed flowering eventually resulting in a higher yield under stress condition. Using F_7_ RILs originating from cross ‘S99-2281 × PI-408105A’ at an early reproductive stage, two QTLs were recently identified and mapped on Chromosome 11 (FTS-11), as well as 13 (FTS-13); these QTLs were related to flood injury score and flood yield index. The significant QTL FTS-13 was reported to be linked to partial resistance to *P. sojae*, with an R2 of up to 18.3%, observed at several locations and years [104]. This provides definite evidence of the link between soybean flooding tolerance and *P. sojae* resistance. It implies that adding flooding-tolerance characteristics would boost resistance to rots caused by fungi, such as *P. sojae* [104]. The University of Missouri developed three improved germplasm lines of soybean for flood-tolerance through MAS. Under non-stress conditions, these germplasms have yielded a potential of 90% of commercial checks, and in severe flood condition, they were found to produce higher yield of 0.7–1.0 tonnes/hectare than commercial checks [130]. Dhungana et al. [115] reported QTLs linked with flooding stress at the V1–V2 stage of soybean. In this study, a RIL population derived from crossing a drought-susceptible (NTS116) and drought-tolerant (Danbaekkong) soybean cultivar was investigated. Based on composite interval mapping technique, they identified 10 QTLs associated with flood tolerance at the V1–V2 stage of soybean that possibly explained up to 30.7% phenotypic variations and can eventually be instrumental in soybean improvement programs. To summarize, marker-assisted mapping has been successful to some extent in identifying QTLs associated with flooding tolerance in oilseed crops, but more efforts are required to identify major QTLs explaining big phenotypic variance in large populations.

### 2.5. Cold Stress

Cold stress, which may include chilling temperatures (below 20 °C) and/or freezing temperatures (below 0 °C), has a severe influence on plant growth and development and greatly impedes plant spatial dispersion and agricultural production. Cold stress may be generated by either chilling (below 20 °C) or freezing (below 0 °C) conditions. Cold stress may also be caused by chilling temperatures (temperatures below 20 degrees Celsius) or freezing temperatures (temperatures below 0 degrees Celsius). It directly inhibits metabolic processes and has indirect effects in the form of cold-induced osmotic (freezing-induced cellular dehydration and chilling-induced reduction of water absorption), oxidative, and other stresses. Cold stress prevents plants from fully expressing their genetic potential by impeding metabolic activity first hand, while other stressors only do so indirectly. The great majority of plants that can thrive in temperate climates do so due to a process known as cold acclimation, which allows them to gain the capacity to endure temperatures as low as freezing.

The SSR, SNP, and EST markers have been successfully employed in achieving cold tolerance in plants [131,132]. Zhang et al. [133], Shinozaki et al. [134], and Lata and Prasad [135] identified the genes that play critical roles in the process by which plants develop tolerance to cold and osmotic stress. As a consequence of these investigations, as well as the use of molecular markers to build high-density physical and genetic maps of new genes, it is now feasible to enhance genetic diversity for desirable attributes, such as the ability to respond to cold stress. This is made feasible by the fact that molecular markers can now generate high-density maps of new genes [136]. At present, multiple genomics approaches are being employed to create new data through the utilization of genetic maps obtained from diverse *Brassica* and *Arabidopsis* species [137]. The accumulation of expressed sequence tags and single-nucleotide polymorphisms in *Brassica* species is generating critical information on genome polymorphism, as well as sequencing data for all stress-related traits.

Transcriptome adjustments from *Arabidopsis* have been exploited to discover genes related to cold treatment and other forms of stress. According to the findings, thirty percent of transcriptomes indicated sensitivity to regulation to common stress, with the majority clearly responding to particular stimuli [138]. In the first organ-specific cDNA fluorescence microarray investigation to evaluate coordinated transcriptional shifts in response to chilling and salinity stress in cultivated sunflower, Fernandez et al. [108] reported that eighty genes were found to be candidate genes for the early response of sunflowers to low temperature and salt stress. Microarray profiling of chilled and NaCl-treated sunflower leaves by the authors revealed dynamic shifts in the abundance of transcripts, including transcription factors, proteins involved in defense and stress, and effectors of homeostasis, all of which emphasize the complexity of both stress responses.

In a similar study, nylon microarrays with more than 8000 putative unigenes was performed to evaluate the transcriptional profiles of two accessions of sunflower viz. Santiago II and Melody, with differential growth rate ability under low temperatures. The results showed that, between the plants developed at low temperature (15 °C and 7 °C) and the corresponding control plants at two-leaf and four-leaf stages, 108 cDNA clones were found to be differentially expressed across the two genotypes with a *p* value of 10^−3^ [107]. Around 90% of these genes, including those involved in protein biosynthesis, signal transduction, and energy, as well as carbohydrate metabolism and transport, were downregulated. Only four genes were identified as being differentially expressed in both genotypes, which further suggests that the response of sunflower plants to these temperature regimes is driven by identical genetic processes. The authors came to the conclusion that the vulnerability of sunflower to cold stress may be caused by the downregulation and/or non-induction of genes playing a vital role in cold tolerance.

Another research group utilized 104 RILs (F_6_-derived lines) of soybean resulting from a cross ‘Hayahikari (chilling-tolerant cultivar) × Toyomusume (chilling-sensitive cultivar)’ in order to identify the QTL linked to freezing tolerance during reproductive stage. This was done to identify the QTL linked with soybean frost resistance during reproductive development. After conducting a genotypic evaluation of the population with 181 markers and correlating genotypic data with seed yield in two different conditions, i.e., chilling and optimal temperature, the researchers were able to identify three QTLs related to freezing tolerance on the basis of seed-yielding ability. These quantitative trait loci (QTLs) were essential for the plant’s ability to withstand cold temperatures. Among these, qCTTSW1 and qCTTSW2 were found to be in close proximity to a QTL for flowering time. It was observed that qCTTSW2 interacted epistatically with a marker locus next to a second QTL for flowering time [109]. In fact, no significant QTL for cold tolerance was detected. An F_2_ generation descended from a cross ‘Hayahikari’ × ‘RIL of Hayahikari’ demonstrated that qCTTSW1 was mostly independent of flowering time. The third quantitative trait locus, qCTTSW3, has been shown to influence chilling tolerance. Another research employed a BC_2_F_3_ population derived from Harosoy (donor parent) × Hongfeng 11 (recurrent parent) to screen soybeans during the germination stage for drought and low-temperature conditions [110]. This population was screened for low-temperature and high-humidity circumstances. The objective of this study was to obtain a deeper knowledge of the genetic overlap between drought and low-temperature-tolerance QTLs in soybean during germination. There is a genetic overlap between drought and low-temperature tolerance during germination, as indicated by the identification of twelve QTLs in soybean that were associated with both drought and low-temperature tolerance. This fact was corroborated by the ability of tolerant soybean to withstand both low temperatures and drought at the same time. On the other side, it was observed that 18 QTLs were associated with drought resistance and that 23 QTLs were associated with cold-temperature resistance. A study was conducted utilizing QTL analysis of seed-yielding capacity at low temperature in soybean, simulated climatic conditions at normal and low temperatures, and RILs obtained from the cross of two cultivars with differing chilling tolerances [111]. The aim of this study was to understand the genetic basis of freezing tolerance and to identify associated genomic regions.

In close proximity to marker Sat 162 at linkage group A2, a quantitative trait locus (QTL) with a substantial effect was identified (LOD more than 15, r2 larger than 0.3). This QTL was shown to be connected with the capacity to generate seeds only at low temperatures. A population of segregating varied inbred families that generated basically identical lineages gave further confirmation of the QTL’s significance. This proof of ancestry was provided by the community of inbred families. It was further reported that the genomic region containing the QTL also influenced the node and pod numbers regardless of temperature condition, although this effect was not primarily associated with chilling tolerance. In summary, several studies successfully demonstrated the association of physiological traits with multiple QTLs (including major QTLs) in oilseed crops under chilling stress and can be a great boon for development of tolerant cultivars in the future.

### 2.6. Heat Stress

**Figure 5:**
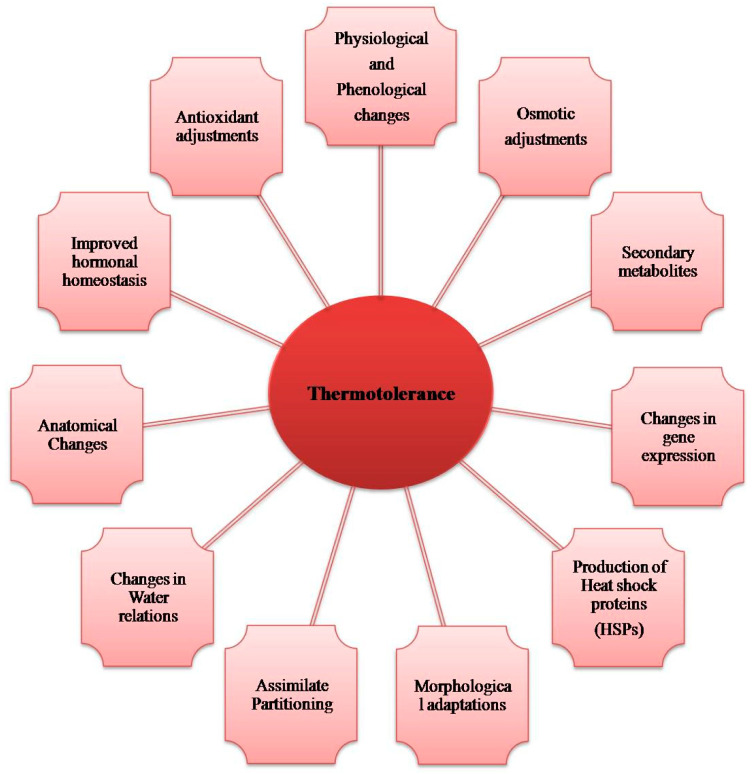
Plant responses to heat stress.

One of the major abiotic factors that lower crop productivity is heat stress. More frequent heat waves are predicted to occur and with greater severity as a result of global warming, aggravating the existing conditions. Therefore, it is crucial to understand the molecular processes that increase crop plants’ tolerance to heat, especially in their reproductive organs. For the manipulation and exploration of pertinent genes for application in crop development initiatives, precise molecular knowledge will be helpful. This can be accomplished by gaining an in-depth understanding of various plant responses to heat stress, deciphering mechanisms of heat tolerance, and developing potential interventions for improving heat tolerance. Reduced photosynthesis, increased photorespiration, decreased availability of water, loss of cell membrane integrity and function, generation of ROS, and many other detrimental impacts are all driven by heat stress. Plants deploy numerous defense mechanisms to combat heat stress, including the increased expression of different enzymatic and non-enzymatic antioxidants to scavenge ROS, maintaining membrane stability, the production of various compatible solutes and metabolites, and the activation of various signaling cascades (Figure 5). Understanding each of these mechanisms will enable us to develop transgenic, traditional, and molecular breeding methods to enhance plant heat tolerance [139]. Numerous studies have documented adverse impacts of high-temperature stress in oilseed crops, such as decreased pollen germination and pollen tube length, which led to pollen mortality and fruit setting in *Brassica* as a result of heat stress [140]., reduction in soybean yield at temperatures more than 26/20 °C [141], and a reduction in seed weight in soybean due to a rise in temperature from 30/25 °C (day/night) during the seed filling period [142].

Considering the uncontrolled nature of environmental factors and the influence of additional biotic stresses, selection for thermo-tolerance through conventional breeding could be extremely challenging. Better techniques are therefore required for carrying out more precise greenhouse tests. Over the past ten years, scientists have turned to various methods to find the genes and QTLs linked with heat stress tolerance. A foundation for identifying the precise chromosomal position of QTLs responsible for plant heat tolerance is currently being laid by breakthroughs in genotyping assays and marker identification [143].

In oilseed crops, several major or minor QTLs and related markers for heat tolerance have been identified, including in peanuts [144], sesame [145], and soybean [146]. Genome maps and molecular markers for major oilseed crops have been identified by many researchers [145,147,148,149,150]. Similar to this, various oilseed crops, including soybean, [151], rapeseed [152,153], cotton [154], sunflower [155], groundnut [156], and sesame [157], are using the genome-wide association mapping technique under heat stress. In a thermo-sensitive dominant genic male sterility (GMS)-based inbred line (TE5A), which originated through the spontaneous mutation of *Brassica napus*, Zeng et al. [112], reported the fine mapping of *BntsMs* (dominant thermo-sensitive GMS gene) using AFLP and intron polymorphism (IP) methodologies. The five AFLP markers associated with the *BntsMs* gene were found by the authors after screening with 1024 primer combinations; two of these markers were then transformed to SCAR markers. Two SCAR markers were found flanking the *BntsMs* gene at a distance of 3.5 and 4.8 cm after studying a sizable BC_2_ population of 700 recessive-fertility lines. Additionally, seven IP markers were also developed and used on the aforesaid population; two of these markers, IP004 and IP470, were placed at a distance of 0.3 and 0.2 cm, respectively, from the flanking region of the *BntsMs* gene.

## 3. Advent of a New Era for Development of Molecular Markers

With the advent of next-generation DNA sequencing technologies, including whole-genome sequencing (WGS), have opened new avenues for a comprehensive overview of the genetic diversity of oilseed crops and their genetic architecture in last decade. More recent advances involving expressed sequence tags (ESTs) also aided genome annotation and could further boost the molecular breeding program. Decent attempts that have been made in the genome sequencing of major oilseeds, such as sesame [158,159], safflower [160,161], rapeseed [162], mustard [163], sunflower [164,165], castor [166,167], flax seed [168], and peanut [169], have revolutionized the development of advanced co-dominant markers, such as SSR and SNPs, for the molecular mapping of abiotic resilience in oilseed crops. Soybean was sequenced through advanced high throughput technology in 2010 [170], and much progress has been made in this oilseed crop relevant to molecular breeding program for the achievement of abiotic stress tolerance and has been fairly covered in a review by Arya et al. [45]. WGS can provide detailed information about the genes associated with crucial and/or complex traits such as yield, oil content, and abiotic stress resistance, thus allowing for more precise selection of cultivars with desirable traits. Indeed, a novel method for SNP detection and mapping carry a huge potential to overcome the limitations of traditional MAS but are still far from being cost-efficient for the marker-assisted breeding of large populations [171]. Overall, WGS capacitates a system breeding approach with molecular markers that can be coupled with high-throughput phenotypic evaluation; such an approach has a potential to integrate gene function information with the improved field performance of oilseed crops.

## 4. Conclusions

Abiotic stresses have significant effect on the growth parameters of oilseed crops due to global climate change. Breeders of oilseeds should design their breeding programme to account for climate change and breed oilseed cultivars that are resilient to the changing climate. Developing reliable markers, which can be employed for different populations, could further enhance selection efficiency for breeding and could be a great milestone for breeding programs. The strong linkage of molecular markers to the desired attribute necessitates that they allow for preferred genotype selection. Abiotic stress tolerance in oilseed crops has also been established using emerging technologies like high-throughput marker systems and marker-based selection approaches, but their use is still limited. Not much work on MAS in this direction is being conducted. MAS is a highly promising strategy to achieve stress tolerance against abiotic stresses. Before beginning a breeding program, genetic diversity can also be assessed using molecular markers. In fact, several QTLs for economic features have already been reported. The use of a large sample size or the construction of multiple biparental cross populations could be useful to map rare alleles. To increase oilseed productivity, efforts should be made to use molecular breeding techniques, which can be expedited by current advancements in next-generation sequencing. The contemporary trend is to combine QTL mapping with the functional genomics methods (like ESTs and microarray) for gene expression studies that can be used to develop markers from genes itself [172]. This technique, called the “candidate gene approach”, holds great potential in identifying the actual gene that controls the trait of interest. These methods can also be used to recognize SNP markers. The development of SNPs and EST-based markers has provided researchers a great tool for QTL mapping and MAS. Moreover, significant progress is being made in QTL mapping between related species through comparative mapping. To reduce the unfavorable effects of various abiotic stresses on oilseeds that are linked to climate change, modern molecular marker technologies must be adopted with traditional breeding techniques to create cultivars resistant to climatic change.

## Figures and Tables

**Table 1:** Yield loss reported in oilseeds due to abiotic stresses.

S.No.	Crop	Abiotic Stress	Yield Reduction	References
1.	Mustard	Moisture stress	17–94%	[5]
2.	Mustard	Salinity	50–90%	[6]
3.	Mustard	Heat stress	34%	[7]
4.	Mustard	Heat stress	>54%	[8]
5.	Soybean	Drought	73–82%	[9]
6.	Soybean	Drought	50%	[10]
7.	Soybean	Salinity	Up to 40%	[11]
8.	Soybean	Flooding	Up to 25%	[12]
9.	Soybean	Flooding	20–39%	[13]
10.	Soybean	Cold stress	24%	[14]
11.	Sunflower	Drought	Up to 40%	[15,16]
12.	Sesame	Waterlogging	50–90%	[17,18]
13.	Sesame	Drought	28%	[19,20]
14.	Safflower	Drought	17.2%	[21]
15.	Groundnut	Drought	55–72%	[22]

**Table 2:** List of studies involving MAS for improvement of abiotic stress resistance in oilseeds.

Mapping Population/Genotypes	Trait	Marker Used/Markers Linked to QTL	Crop	Reference
Hutcheson × PI471938, 140 F_4_ population	Drought tolerance	SSR (Satt226)	Soybean	[97]
Jackson × KS4895, 81 RILs	Drought tolerance	SSR (Sat_044)	Soybean	[98]
Minsoy × Noir 1, 236 RILs	Drought tolerance	SSR (Satt205-Satt489)	Soybean	[99]
S-100 × Tokyo, 116 F_2_ population	Drought tolerance	RFLP (A489H)	Soybean	[25]
Young×PI416937, 120 F_4_ population	Drought tolerance	RFLP (B031-1, A089-1, cr497-1, K375-1, A063-1)	Soybean	[47]
TAG 24 × ICGV 86031, RILs	Drought tolerance	SSRs	Groundnut	[61]
ICGS 76 × CSMG 84-1 and ICGS 44 × ICGS 76, RILs	Drought tolerance	SSRs	Groundnut	[62]
Mex.22-191 × IL.111 F_3_ population	Drought tolerance	SSRs and ISSRs	Safflower	[57]
400 accessions including landraces and modern cultivars	Drought tolerance	SNPs	Sesame	[66]
S-100 × Tokyo RILs	Salt stress	SSRs (Sat_091)	Soybean	[86]
FT-Abyara × C01 and Jindou No. 690197, RILs	Salt stress	SSRs (Sat_091)	Soybean	[87]
Kefeng No. 1 × Nannong1138-2, RILs	Salt stress	SSRs (Sat_164 and Sat_358)	Soybean	[88]
Jackson (PI548657) × JWS156-1, F_2_	Salt stress	SSRs	Soybean	[89]
490 accessions	Drought and salt stress	SNPs	Sesame	[68]
AC Hime × Westag-97, RILs	Cadmium toxicity	SSRs (SatK147, SacK149, and SattK152)	Soybean	[100]
Essex × Forest, RILs	Manganese toxicity	SSRs (Satt291, Satt239, OEO2)	Soybean	[101]
Archer × ‘Minsoy and Archer × Noir I’, RILs	Flooding	SSRs (Sat_064)	Soybean	[102]
Misuzudaizu × Moshidou Gong 503, RILs	Flooding	SSRs	Soybean	[103]
S99-2281 × PI 408105A, RILs	Flooding	SSRs and SNPs (Sct_033, BARC-024569-04982, BARC-016279-02316)	Soybean	[104]
A5403 × Archer (Population 1) × P9641 × Archer (Population 2), F_6:11_ RILs	Flooding	SSRs (Satt385, Satt269, Sat_064)	Soybean	[105,106]
Santiago II and Melody	Cold stress	ESTs	Sunflower	[107]
HA89	Cold stress	ESTs	Sunflower	[108]
Hayahikari × Toyomusume, RILs	Cold stress	SSRs	Soybean	[109]
Hongfeng11 × Harosoy, BC_2_F_3_	Cold stress	SSRs	Soybean	[110]
RILs	Cold stress	SSRs	Soybean	[111]
TE5A, BC_2_	Heat stress	AFLPs, SCARs	Rapeseed	[112]
Jinhuangma (JHM) and Zhushanbai (ZSB), landraces	Drought stress	SNPs	Sesame	[71]
RILs (Per × R500) and DH lines (Major × Stellar)	Cold stress	RFLPs, AFLPs	Mustard and canola	[113]
Mex.22−191 × Goldasht, F_9_ RILs	Drought	AFLPs	safflower	[114]
K099 × Fendou 16, F_7_ RILs	Drought	SSR (Sat_165 and Satt621)	Soybean	[46]
NTS116 × Danbaekkong, RILs	Flooding	SNPs	Soybean	[115]

## Data Availability

No new data were created or analyzed in this study. Data sharing is not applicable to this article.
